# CASE REPORT Reconstruction and Characterization of Composite Mandibular Defects Requiring Double Skin Paddle Fibular Free Flaps

**Published:** 2013-04-26

**Authors:** Austin M. Badeau, Frederic W.-B. Deleyiannis

**Affiliations:** ^a^University of Colorado School of Medicine, Anschutz Medical Campus; ^b^University of Colorado Hospital & Children's Hospital Colorado, Division of Plastic and Reconstructive Surgery, Departments of Surgery and Otolaryngology, Aurora, Colo

## Abstract

**Objective:** Fibular free flaps are the preferred method for reconstruction of composite lateral mandibular defects. This reconstructive technique is limited by the skin paddle's inability to freely rotate when attempting to fill 2 poorly aligned defects. Reconstructive surgeons have been exploring multiple methods of creating 2 independent skin paddles based on the same peroneal blood supply. We present a variation of these techniques. **Method:** Our patient with a history of squamous cell carcinoma presented with a left retromolar recurrence and osteoradionecrosis of the mandible with a draining anterior sinus tract. The combination of these defects warranted further composite resection with fibular free flap reconstruction. **Results:** A subperiosteal dissection was performed to create 2 separate septocutaneous skin paddles based on the same peroneal blood supply. This dissection and discard of proximal fibula provided the rotational freedom needed for the 2 skin islands to fill both a lateral oral defect and anterior cutaneous defect. **Conclusion:** Although similar reconstructive methods have been reported in the literature, the characterization of defects benefiting from these techniques is scarce and unclear. We describe clear and concise characteristics of these defects, which should be meaningful to the reconstructive surgeon when considering operative technique.

Reconstruction of oromandibular defects poses unique challenges to the reconstructive surgeon. Surgical technique and ideal flap choice have been debated ever since flap repair became favored over split-thickness skin grafts for lateral mandibulectomy repair in the mid 1990s.^1^ Osteocutaneous fibular free flaps are currently accepted as the preferred method for reconstruction of composite through-and-through mandibular defects. This flap uses a large skin paddle to cover both the intraoral and cutaneous defects by draping 2 skin islands, which are separated by de-epithelialized tissue over the inset fibula.^2^^,^^3^ This method of reconstruction requires that the intraoral and cutaneous defect be similar and well aligned, as the skin islands used to fill these defects have little freedom to rotate and/or translate independently of each other. Previously described techniques have relied on 2 separate flaps to fill poorly aligned defects; one osteocutaneous flap (eg, fibular free flap or osteocutaneous radial forearm flap) for the oromandibular defect and another soft tissue flap (eg, pedicled or free).^4^ However, there have also been various reports describing surgical techniques used to fix “complex” mandibular defects using 2 independent skin paddles based on the same peroneal blood supply.^5^^-^6 This article describes another surgical technique for repairing a “complex” lateral mandibular defect using a double skin paddle fibular free flap and further characterizes the defect in which this type of repair should be considered.

## METHOD/CASE PRESENTATION

The patient was a 59-year-old gentleman with a history of oropharyngeal cancer originally diagnosed as left basaloid squamous cell carcinoma (SCC) and treated with definitive radiation and chemotherapy in 2009. In 2011, a recurrence was found, which was treated with a partial glossectomy and tonsillar fossa resection and anterolateral thigh (ALT) free flap reconstruction. During the intervening year, he developed osteoradionecrosis (ORN), which was managed with antibiotics and hyperbaric oxygen therapy. At the same time, he developed an anterior submental draining sinus tract extending to the bone. Most recently, he was found to have a left retromolar SCC mass grossly extending into the ALT flap. Given the patient's SCC recurrence and ORN, further composite resection with fibular free flap reconstruction was recommended.

## RESULTS

**Figure 1:**
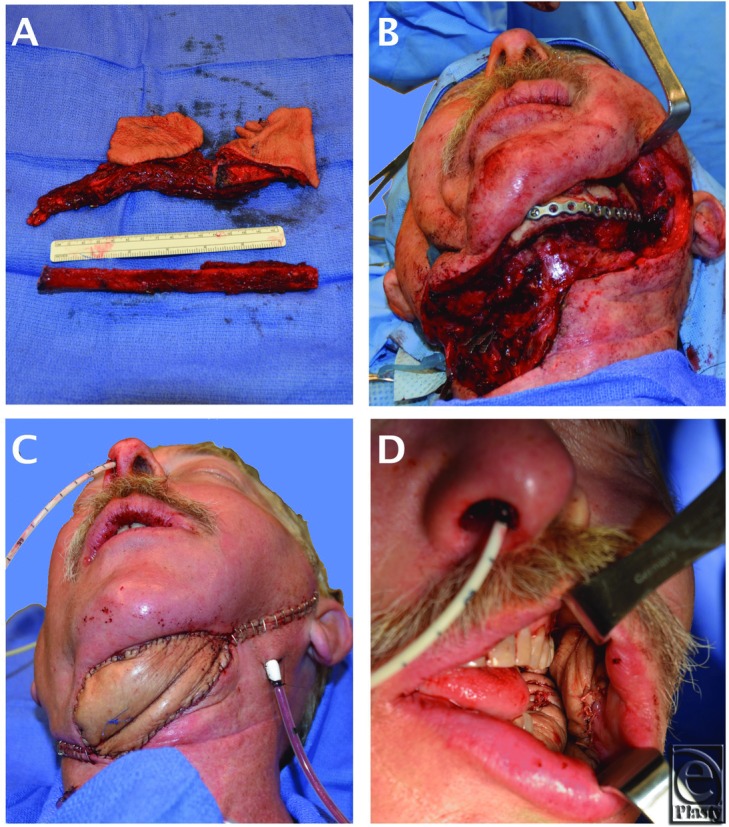
(*a*) Double skin paddle fibula free flap freed from fibula. (*b*) Dissection showing lateral mandibular and submental defect. (*c*) Flap inset into submental defect. (*d*) Flap inset into oral defect.

**Figure 2:**
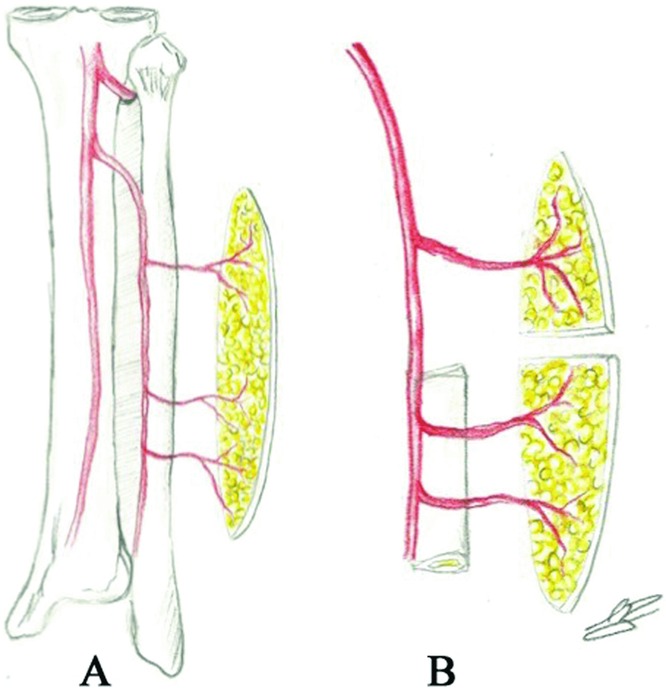
(*a*) Dissected skin paddle of fibula free flap. (*b*) Two independent skin paddles created after subperiosteal dissection and discard of proximal fibula graft.

The patient underwent a composite resection, which included the oral tumor, the lateral mandibular body, the anterior sinus tract and the inflamed submental skin (Fig 1b), and a portion of the previous ALT flap. A large osteocutaneous fibula free flap was designed and harvested from the patient's right lower leg. The skin paddle was approximately 15 cm in length × 8 in width oriented along the longitudinal axis of the fibula. Three septocutaneous perforators were identified and captured approximately 7 cm, 9 cm, and 15 cm proximal to the lateral malleolus. Osteotomies were performed 6-cm distal to the fibular head and 6-cm proximal to the lateral malleolus. Two separate skin islands were then created from the paddle by performing a subperiosteal dissection of the proximal fibula and dividing the posterior lateral septum and skin between the proximal 2 perforators (Fig 2a). The proximal fibula was discarded (Figs 1a and 2b). By doing so, the 2 skin islands were now free to rotate independently of each other, allowing for cutaneous closure of the submental defect and osteocutaneous reconstruction of the lateral composite defect (Figs 1c and 1d).

## DISCUSSION

Ongoing research into the vascular supply of the lower leg will continue to help define the possibilities of lower leg free flaps. The literature has shown that the lateral leg possesses a rich and reliable cutaneous blood supply including musculocutaneous perforators, which predominate in the proximal two-thirds of the lateral lower leg and septocutaneous perforators, which predominate in the distal two thirds of the lower lateral leg.^7^ Heitmann et al^8^ demonstrated an average of 4.8 cutaneous peroneal perforators per leg with 66% of these being septocutaneous perforators and 34% musculocutaneous. These data are in agreement with Beppu et al,^9^ who found 62% of cutaneous perforators to be of septal origin.

The improved understanding of the vascular anatomy of the lateral leg^8^^-^9 has allowed surgeons to confidently explore multiple techniques for acquiring 2 independent skin paddles from the same peroneal vascular supply, thus eliminating the need for 2 separate free flaps. Daya^6^ described a “chimeric flap” composed of a peroneal artery musculocutaneous perforator flap and a composite fibula osteoseptocutaneous flap to fill a large anterior mandibular defect with complex 3-dimensional anatomy. Yang et al^5^ described the use of a double skin paddle peroneal tissue transfer fed by 2 distinct septocutaneous perforators or even 2 branches of the same septocutaneous perforator to fill large through-and-through mandibular defects. Finally, our reconstruction utilized septocutaneous peroneal perforators to create a distal composite osteocutaneous flap and a proximal cutaneous flap.

All of these reconstructive options create 2 skin paddles capable of independent rotation while avoiding the disadvantage of additional anastomoses required of 2 separate free flaps and the comorbidities related to a second donor site. The reconstructive technique described by Daya^6^ offers the possibility of a very large proximal skin paddle and the long pedicle of this skin paddle offers a high degree of independent movement. Daya^6^ also asserts that a flap based on musculocutaneous perforators is easier to raise than flaps based on septocutaneous perforators because of their close adherence to the periosteum. During our case, we found a subperiosteal dissection to be a reliable way of raising and preserving the septocutaneous perforators.

A full investigation into the advantages and disadvantages of each of the aforementioned techniques needs to be further researched. However, before this can happen, the type(s) of defect requiring these reconstructive methods needs to be further characterized. Despite reports dating back to 2000,^5^ the use of independent double skin paddle fibular free flaps for mandibular reconstruction is still an unfamiliar concept. Perhaps this is because the defects requiring such reconstruction are fairly rare, but the language used to describe the indications for these surgical techniques has been inconsistent and vague as well. Previous studies have used terms like “complex,” “large,” and “different spatial orientation” to characterize the defects. These terms are neither descriptive nor specific enough to understand the indications for choosing a fibular free flap reconstruction with 2 independent skin paddles.

**Figure 3:**
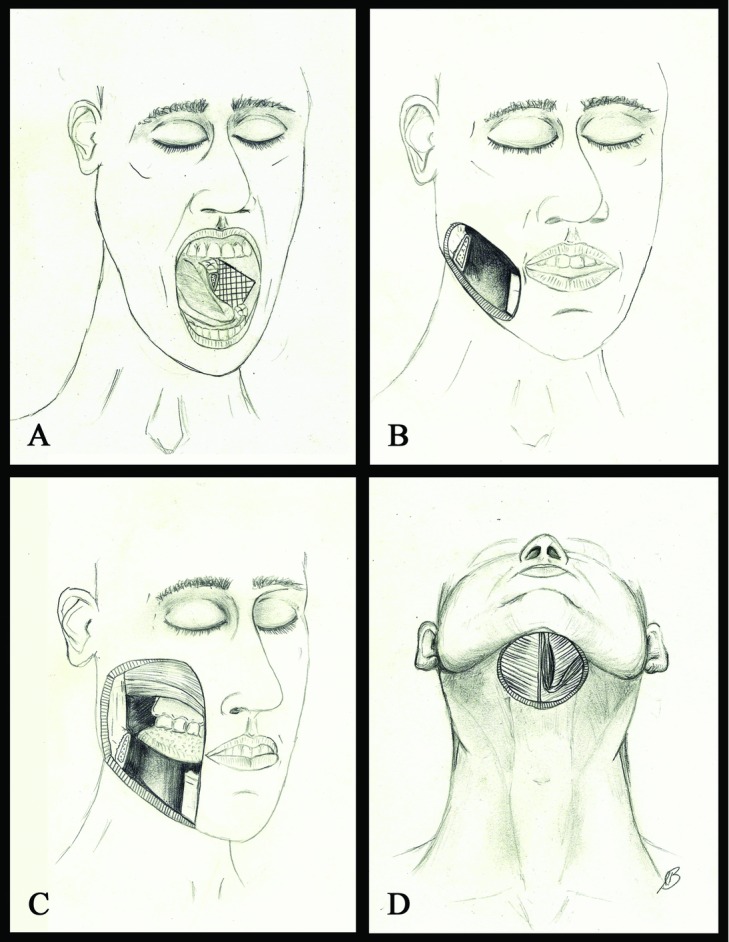
(*a*) “Type 1” defect with resection limited to the lateral mandible and soft tissue of the oral cavity (i.e., floor of mouth, lateral tongue). (*b*) “Type 2” through-and-though defect involving the lateral mandible, oral cavity, and the skin overlying the mandible. (*c*) “Type 3” through-and-through defect with a large volume soft tissue resection with the soft tissue resection above the plane of the mandible (ie, cheek). (*d*) “Type 4” defect with submental cutaneous resection and a concurrent noncontiguous/noncongruent oral cavity defect as seen in 3A.

Previous studies have proposed a classification system for lateral mandibulectomy defects as an attempt to produce an algorithm for reconstruction.^1^ Type 1 lateral defects were characterized by soft tissue resection limited to the oral cavity. Type 2 defects included soft tissue resection of both the oral cavity and skin atop the lower one third of the face or neck creating a through-and-through defect. Type 3 defects were characterized by through-and-through defects with large volume/area soft tissue resection of the skin overlying the midface, parotid, and/or cheek. Each type of defect lends itself to a preferred type(s) of flap reconstruction. We propose characterizing a “Type 4” defect for which fibular free flap reconstruction with 2 independent skin paddles should be considered (Figs 3a-3d).

“Type 4” defects can be described as lacking one or both of the following properties: congruence and contiguity. Geometrical congruence is defined as 2 objects having the same size and/or shape. Contiguity, or the state of being contiguous, can be described as objects that are adjacent or sharing an edge/border. Lacking these properties, “Type 4” defects are most aptly described as being either noncongruent and/or noncontiguous.

Noncongruent defects could include a round oral defect with a square cutaneous defect or a small oral defect with a very large cutaneous defect. In either case, the rotational freedom provided by 2 separate skin paddles would allow for better individualization and insetting of the flaps into the defect as compared to folding a single (de-epithelialized) skin paddle over itself and the inset fibula.

A noncontiguous defect could include a posterior-lateral oromandibular defect with a contralateral neck defect. Even if these 2 defects were congruent, their lack of contiguity prevents reconstruction with a single skin paddle. A fibula free flap with 2 independent skin paddles provides the high degree of rotational and translational movement necessary to reconstruct such a defect using a single free flap.

Though relatively uncommon, noncongruent, and noncontiguous defects can arise from a number of different pathologies including, but not limited to, traumatic blast/gunshot wounds to the head and neck, oropharyngeal cancer with disproportionate cutaneous involvement, and in our case ORN leading to an anterior draining sinus tract.

The reconstructive challenges of oromandibular defects have been described in the literature with a variety of proposed reconstructive solutions. Improved understanding of the vascular anatomy of the lower leg has demonstrated that osteocutaneous fibular free flaps with 2 independent skin paddles can be reliably harvested. Noncontiguous and noncongruent oromandibular and cutaneous defects represent ideal indications for double skin paddle fibular free flap reconstruction if independent peroneal perforators can be captured in the flap design.
